# Approaches for Measuring Inclusive Demographics Across Youth Enjoy Science Cancer Research Training Programs

**DOI:** 10.15695/jstem/v5i2.12

**Published:** 2023-01-23

**Authors:** Megan A. Mekinda, Sunita Chaudhary, Nathan L. Vanderford, Karen Burns White, Linda S. Kennedy, Lisa K. Marriott

**Affiliations:** 1 University of Chicago Medicine Comprehensive Cancer Center, Chicago, IL; 2 Rutgers Cancer Institute of New Jersey, Department of Surgery, Robert Wood Johnson Medical School, New Brunswick, NJ; 3 Markey Cancer Center and the Department of Toxicology and Cancer Biology, College of Medicine, University of Kentucky, Lexington, KY; 4 Dana-Farber/Harvard Cancer Center, Boston, MA; 5 Dartmouth Cancer Center, Geisel School of Medicine, Hanover, NH; 6 OHSU-PSU School of Public Health; Oregon Health and Science University; Portland, OR

**Keywords:** Health Inequities, Historically Underrepresented, Biomedical Workforce Development, Undergraduate, High School

## Abstract

The National Cancer Institute’s (NCI) Youth Enjoy Science Program (YES) funds initiatives to support the cancer research training and career ambitions of middle school through undergraduate students from populations underrepresented in the biomedical sciences. The program has funded 16 institutions nationally as of January 2022. Given the program’s focus on increasing diversity within the cancer research workforce, demographic characteristics of YES trainees provide essential information about the populations being served and program effectiveness. Six programs formed an interest group focused on trainee demographics and surveyed all YES grantees about their demographic data practices. Fifteen programs (94%) completed the survey. Survey data were analyzed through descriptive statistics and thematic coding. Findings revealed considerable variability in programs’ approach to demographic data, including which demographics were measured, how they were operationalized, and when and how the data were collected. Half of YES programs (53%) could report underrepresented populations in biomedical research among trainees using consistent definitions. Most programs described efforts to improve their demographic data practices; however, challenges remained for the vast majority. In consideration of these findings, we offer recommendations for inclusive demographic data practices to better define and retain underrepresented populations in biomedical sciences.

## INTRODUCTION

Cancer does not discriminate, although within the United States, the burden of cancer incidence and mortality is not shared equally among demographic groups. Cancer health inequities are documented among historically underrepresented racial and ethnic populations (Zavala et al., 2021), rural populations (Rodriguez et al., 2018), low socioeconomic populations (Lortet-Tieulent et al., 2020) as well as sexual and gender minorities (Haviland et al., 2020; Kano et al., 2020). Access to cancer care and treatment can vary widely in the United States, particularly for individuals living with disabilities (The Uancet Oncology, 2019) and those from lower socioeconomic groups. Unequal access to cancer clinical trials can perpetuate cancer health inequities (Sharrocks et al., 2014), depriving some populations of novel, potentially more effective treatments and undermining researchers’ knowledge of differences in outcomes across diverse patient groups. Accordingly, the National Institutes of Health (NIH) declared “increasing diversity in research participation, particularly among those who are underrepresented in clinical trials” a component of its strategic plan to address the nation’s most pressing health needs (National Institutes of Health, 2021b, p. 15).

One potentially powerful strategy to enhance clinical trial recruitment and, ultimately, to improve health equity, is increasing the diversity of the national cancer research workforce; that is, engaging members of the very populations facing cancer inequities in the development of science to solve cancer problems. Early-intervention research training programs can provide critical professional development support for youth from historically underrepresented groups and motivate them to pursue careers in science, technology, engineering and mathematics (STEM) (Carpi et al., 2017; Hunter et al., 2007; Thiry et al., 2011).

The National Cancer Institute’s (NCI) Youth Enjoy Science Program (YES) funds initiatives to support the cancer research training and career ambitions of middle school, high school, and undergraduate students from populations underrepresented in the biomedical sciences (National Institutes of Health, 2016). The program has funded 16 institutions nationally as of January 2022. Given the program’s focus on increasing diversity within the cancer research workforce, demographic characteristics of YES trainees provide essential information for understanding the populations being served and evaluating program effectiveness. YES programs largely determine what demographic data to collect as well as approaches for data collection, analysis and interpretation. NIH inclusion tables document participants in biomedical research and include gender, race, and ethnicity (U.S. Department of Health and Human Services, 2012). Historical scales for such demographic variables are often narrow – for example, binary measurement of gender as male or female – and do not fully capture lived experiences of a wide variety of individuals from underrepresented populations (Morrison et al., 2021). Furthermore, NIH’s interest in increasing diversity includes measurement of demographics related to disability and disadvantaged backgrounds, which are also populations historically underrepresented in research careers (National Institutes of Health, 2019). Many training program providers collect data on other intersectional metrics that, similar to demographic categories, may predict trainees’ pursuance of a cancer-related career (e.g., grade point average, degree attainment and research publications).

This manuscript assessed the demographic data practices of YES training programs to understand the range of data captured and their utility in optimizing trainee outcomes. The purpose was to facilitate effective comparison of data across programs, a first step toward determining the extent to which programs are collectively meeting goals for increased diversity within the cancer research workforce.

## METHODS

### Participants and Settings.

In October 2020, the NIH Center to Reduce Cancer Health Disparities hosted a four-day virtual conference for R25 principal investigators and their staff. The purpose was to share best practices within YES programs and facilitate multi-institutional collaborations to enhance trainees’ experiences and outcomes. As a result of that meeting, leaders from six out of 16 funded YES programs formed an interest group. Their initial goal was to harmonize trainee demographic data collection across programs, facilitating data aggregation and program-to-program comparisons of trainee characteristics and outcomes. Initial discussions revealed a variety of demographic data practices among interest group participants. To examine the full range of approaches to demographic data, in December 2021, the group surveyed all YES grantees.

Survey invitations were sent via email to the principal investigators of the 16 R25 YES programs, with instruction to have the survey completed by the individual most knowledgeable about their program’s data collection. The survey was developed and administered through REDCap (MAM). Sixteen primary survey items were co-developed by the team and comprised quantitative and qualitative prompts (Appendix A: Survey).

### Survey Measures.

In addition to providing basic identifying information about their institution and grant details (Appendix A), respondents completed a matrix to indicate which categories of trainee demographic data they collect and the timing of collection for each measure. The categories included variables specifically mentioned in the 2019 Notice of the National Institutes of Health’s Interest in Diversity (National Institutes of Health, 2019): Race, ethnicity, first-generation college status, socioeconomic status and disability status. The matrix included five additional variables identified by the group as potentially important to understanding their trainees’ needs, experiences and outcomes: gender, preferred pronouns, sexual orientation, grade point average (GPA), and languages spoken at home. While GPA and languages spoken are not generally considered demographic characteristics, they are reported here as they can provide important contextual information about their intersection with demographics underrepresented in biomedical science. Using open prompt, survey respondents were able to note any demographic categories captured by their program that were not explicitly listed in the matrix.

For each category, respondents marked if the data were collected at time of application, at time of enrollment, at another time (which respondents were asked to specify), or not at all. Branching logic for gender and disability status prompted those who collect this data to share whether they presented gender options beyond the binary and if they used 2019 NIH criteria for defining disadvantaged or disabled populations (National Institutes of Health, 2019).

Additional survey items prompted respondents to elaborate on aspects of their approach to the collection and use of trainee demographic data. Specifically, a series of open-response items asked programs to describe any documentation collected to validate trainees’ self-reported data (e.g., attestation from a financial aid office to verify socioeconomic status); ways in which their team’s approach to demographic data has evolved over the course of their YES program; and current challenges their team faces regarding the collection, interpretation or application of demographic data. A final open-response item invited respondents to share any additional thoughts regarding demographic data practices not covered by previous items.

### Data Analysis.

Data were exported from REDCap into SPSS to analyze descriptive statistics for all nominal data (e.g., frequencies for the categories of demographic data and timing of data collection listed in the matrix). Data from open-ended prompts were exported into Microsoft Excel, where they were thematically coded by two researchers with qualitative research experience (MAM and LKM), one of whom teaches qualitative methods at their university. Coders used columns in Excel to permit co-coding of themes, as necessary. They met virtually and came to agreement on themes (see Appendix B: Coding Dictionary with Data Definitions), which were discussed with co-authors during bi-weekly meetings. Finalized themes, and their frequencies, were summarized into matrices for each survey prompt.

### Confidentiality of Data.

Electronic files were kept on a secure server at the University of Chicago and password-protected. Identities of the survey respondents were known only to the authors. No personal data of students or respondents were captured, including personal health information. Because this case study focused only on educational practices of YES programs, which are typically exempt from Institutional Review Board (IRB) review, IRB approval was not sought. However, ethical practices of voluntary completion and privacy were emphasized to all programs prior to survey participation.

## RESULTS

### Participants and Settings.

Responses were received from all 16 YES programs. Surveys were completed by 15 (94% response rate), with one of the newest YES grantees declining to participate given that they had not yet initiated data collection. Survey respondents averaged 13.3 years of professional experience in the field (SD=6.1; median=15 years) and included six principal investigators (40%) and nine staff or administrators (60%). Programs were funded 1-4 years prior to survey administration, with approximately equal distribution.

### Demographic Data Captured.

A range of demographic-related variables were collected by responding programs (Table 1), with race and ethnicity being the only variables collected by all. Twelve programs asked about gender (80%); 10 of these (83%) provided non-binary response options. Thirteen programs (87%) collected data about trainees’ socioeconomic status (SES). Similarly, a question about trainees’ disability status was asked by 10 of 14 programs (71%), yet only three of these (30%) reported using disability definitions explicitly referenced by the NIH (National Institutes of Health, 2019). Eight of 15 programs (53%) explicitly referenced the Notice of NIH’s Interest in Diversity (2019) or documents that reference it, such as the NCI YES program announcement (National Institutes of Health, 2021a). Ten programs (67%) reported collecting racial/ethnic, disability, and SES data more broadly, though SES data collected may not explicitly match the multi-step criteria currently used to define disadvantaged populations (National Institutes of Health, 2019). Specifically, NIH definitions for disadvantaged background (2019) defines meeting two or more criteria, including experiences of homelessness, foster care, low socioeconomics, residing in underserved geographic areas, and being a first generation college student. Therefore, some programs may be using older definitions for SES and disadvantaged backgrounds (National Institutes of Health, 2016, 2018) rather than the current multi-step criteria defining disadvantaged background (National Institutes of Health, 2019). As a result, the operational definitions used for underrepresented populations may not be comparable across program sites.

Write-in responses (Appendix B) included demographic themes of nationality and affiliations (i.e., citizenship or permanent resident status, tribal affiliation, refugee or immigrant status, veteran status), geography (i.e., mailing address, zip code, urban-rural-frontier designation (Health Resources and Services Administration, 2012; Oregon Office of Rural Health, 2019)), home environment (i.e., single parent or guardian home, parent or guardian employment, student report of family’s medical-related employment data) and age (i.e., date of birth). Some programs also listed data related to trainees’ educational status, such as school enrollments, courses taken, academic transcripts, and classifications based on accrued credit hours, although these items were not considered demographics per se by the study team. Neither were experience-based variables listed by respondents such as family cancer history, a potential proxy for genetic risk of cancer.

### Data Verification.

Almost all programs (14 of 15; 93%) reported collecting documentation to verify information provided by trainees (Appendix B). Of the fourteen programs that asked trainees about first-generation college student status, seven (50%) verified the information provided by trainees by also asking for their parents/guardians’ educational attainment. Only three programs (20%) mentioned verifying information specific to NIH definitions for underrepresented populations, with each using a different methodology including parental attestation of trainees’ demographic classifications, proven eligibility for Title VI Indian Education programs (Anderson et al., 2019) and optional submission of financial documentation (e.g., redacted tax forms or applications for federal student aid).

Beyond demographic data verification, of the 13 programs that collect trainees’ GPA at time of application (Table 1), 12 (92%) also request official or unofficial transcripts (Appendix B). One program reported removing transcripts from their verification procedures due to trainee burden, relying instead on self-reported GPA. Two programs (13%) described processes for triangulating application data using interviews or letters of recommendation, although no specific variables were cited as being verified.

### Program Admissions Factors.

In response to the prompt, “Of the demographic data collected at time of application, which categories factor into your team’s admissions decisions?” 11 of the 15 programs (73%) cited diversity-related variables defined by NIH criteria, particularly race and ethnicity, disability, socioeconomic status, and first-generation college status (National Institutes of Health, 2019). Gender and veteran status were also cited. The remaining four programs (27%) described demographics specifically in the context of their catchment area, targeted recruitment efforts, or qualifying criteria for the program, but not with respect to admissions decisions: “[W]e only consider these categories to see if students qualify (if we can serve them), but they play no other factor in our admission decision.” One program cited “automatic admission for a few close community partners and their students, [including] 1) Deaf and Hard of Hearing Students each year, 2) students from Funding for the Advancement of Minorities through Education (predominately Black students), and 3) Students from the Homeless Children Education Fund (all experience homelessness).” This program further explained that they “monitor the gender, racial, and ethnic breakdown of students before sending acceptance.” Another program reported that “[d]ata is collected during outreach efforts that can identify students living in troubled neighborhoods (i.e., higher crime rates, food deserts, poverty-stricken areas, etc.) through conversations with teachers, program directors, and principals.”

Five programs (33%) described non-demographic items that factor into their admissions decisions, including grades, student essays, and letters of recommendation. One of these programs changed practices based on the intersectionality of GPA with demographic factors: “GPA was removed from admissions decisions based on the pleading of our teacher advisory team. They felt it was inequitable due the unconscious bias that can go into student grading in the first place. Plus, some students have competing demands (family/work obligations) that can interfere with schoolwork due to SES issues more than ability to do the work.”

### Evolution of Demographics.

Themes regarding the evolution of programs’ approaches to the collection and use of demographic data were described by 12 respondents (80%) and summarized in Table 2. Most commonly, respondents reported changes to the type of data collected (8 of 15; 53%), with all eight adding or expanding demographic variables (e.g., preferred pronouns) and two programs (13%) eliminating items, specifically gender. Nine of 15 programs (60%) described changes to their methods of data collection, including (1) adjustments to timing or format (e.g., from word-of-mouth to formalized surveys); (2) revisions to the wording or organization of data collection instruments; or (3) the inclusion of definitions for demographic categories to inform trainees’ self-classifications.

Nine of 15 programs (60%) offered rationale for changes to their demographic data practices. Seven of the 9 respondents (78%) noted efforts to be more inclusive, specifically with regard to the addition or expansion of demographic categories to reflect trainees’ diverse, nuanced, and intersecting identities. Five programs (71%) made changes to incorporate updated social norms, perspectives, and evolving best practices for demographic data collection. Four programs (57%) described efforts to be more responsive to trainees’ needs, and four programs (57%) described changes to better align their program data with the standards established by key stakeholders, including the NIH. Three programs (43%) described efforts to improve data accuracy. Finally, three programs (43%) reported no changes to their approach to trainee demographic data. Programs reporting no changes were in their fifth, third and second year of funding.

### Difficulties.

Eleven of 15 programs (73%) described ongoing difficulties with regard to trainee demographic data, with themes summarized in Table 3. (The remaining four programs did not respond to the prompt.) Most commonly, respondents reported concerns about data integrity (7/11; 64%), specifically that data are incomplete or inaccurate. For example, they wondered if and how their programs should collect information about specific demographic categories, such as disability status and gender identity; whether reliance on trainee self-report resulted in under-reporting of certain demographic groups, such as first-generation college students; and whether the methods or timing of data collection undermined trainees’ confidence in disclosing sensitive information, resulting in non-reporting.

Five of 11 responding programs (45%) had concerns about protecting trainees’ privacy and their sense of dignity or self-respect, given the sensitive nature of demographic data. Respondents described data collection as potentially invasive, or suggestive that trainees are somehow lacking. As one respondent wrote, “Students have no desire to be known as a ‘have not’…so we don’t embarrass them by making them answer these types of questions.” Privacy concerns were often presented in juxtaposition to the desire for complete data, suggesting perceived tension between the two.

Four programs (36%) described challenges related to data analysis and reporting. In particular, respondents noted that efforts to align demographic data practices with evolving conventions can make it difficult to reconcile data from one trainee cohort to the next. Respondents also identified shortcomings in templates for data reporting, specifically those that reduce individual trainees to a single demographic category, such as “mixed race.” One respondent cited data showing that the practice results in underreporting of racial and ethnic groups; another asserted, “This is fully against all we do.”

Three programs (27%) reported concerns with data utility, or the extent to which demographic information collected can facilitate program implementation or enhance trainees’ experiences and outcomes. For example, one respondent explained that “Asking for pronouns rather than specific gender has sometimes made it difficult to gauge whether or not we might have even numbers of male and female students,” information needed to comply with their institution’s campus housing guidelines. Another respondent described insufficient bandwidth to analyze and interpret demographic data intended to facilitate personalized programming, which undermined their rationale for collecting the data in the first place.

### Resources for Informing Demographic Data Collection.

When prompted, eight of the 15 respondents (53%) shared citations or links for resources that have informed their demographic data practices (Appendix B). Seven of the eight (88%) cited NIH’s Notice of Diversity (National Institutes of Health, 2019). Other citations included the YES program announcement (National Institutes of Health, 2016) as well as resources for measuring race and ethnicity (e.g., McGee, 2020; Oregon Office of Rural Health, 2019), disability (Americans with Disabilities Act, 2009; McGee, 2020), and socioeconomics for TRIO eligibility using U.S. Census definitions for low income (Office of Postsecondary Education, 2021). Two programs (25%) cited literature for inclusive approaches for demographic data collection (Fernandez et al., 2016) in the context of career expectancy (Metz et al., 2009), with one program developing a public-facing website highlighting approaches for measuring inclusive demographics in the context of STEM and biomedical training research (Paris et al., 2021). Seven of 15 survey respondents (47%) did not respond to the prompt.

## DISCUSSION

Inclusive and accurate measure of trainees’ demographic characteristics is essential to evaluate whether the YES program enhances diversity within the cancer research workforce. It can also inform the development of program activities and experiences sensitive to trainees’ unique needs. Yet, this case study of YES programs revealed considerable variability in programs’ approach to demographic data, including which demographics were measured (Table 1), how they were operationalized, and when and how the data were collected. Half of YES programs (8/15; 53%) could report underrepresented populations in biomedical research among trainees using consistent definitions (National Institutes of Health, 2019), reflecting considerations associated with language, semantics, and training. Specifically, most programs measured demographic data of their participants in some way, including race (100%), ethnicity (100%), disability (71%), as well as aspects of disadvantaged background, including first-generation college student status (93%) and socioeconomic status (87%). However, NIH’s definition of ‘underrepresentation in biomedical sciences’ reflects a composite variable that was revised in 2019 to include seven sub-categories within disadvantaged background (NIH, 2019). Thus, while 14 of 15 (93%) of the programs collected demographic information as well as documentation to verify it, only half (8/15; 53%) inquired specifically about criteria included in the NIH’s definitions for disadvantaged backgrounds, such as experiences of homelessness or foster care (National Institutes of Health, 2019). If a composite variable was broadened to include SES (instead of disadvantaged background), 10 of 15 (67%) measured all three broad categories. Thus, there is operational variability in the demographic data collected by programs, which reflect field advancements in inclusive science for equitable data collection.

Most programs described efforts to improve their demographic data practices over the course of their YES award for the purposes of increasing inclusivity, data accuracy, sensitivity to trainees’ needs and alignment with the NIH and other key stakeholders (Table 2). However, challenges remained for the vast majority of respondents (Table 3), with ongoing concerns about data integrity, trainee privacy, data analysis and reporting, and data utility. Here we discuss these findings and offer recommendations for our YES colleagues and others dedicated to the success of an increasingly diverse and inclusive biomedical research workforce. This case study highlights why supporting inter-fundee communication can advance overall funder goals (Lin, 2022).

### Challenge 1: Complexity of Demographics.

Respondents in this case study represented an average of 13.3 years of professional experience in the field, with their YES programs varying in size and years of funding. Approximately 60% (9/15) of survey respondents had 15+ years of experience and more than one quarter (4/15) had 20+ years of program experience, documenting that these challenges in demographic practices are not limited to new programs.

Programs’ survey responses reflect inherent complexities of demographic data practices. In particular, demographic variables including race, ethnicity, and gender are fluid social constructs. This requires programs’ approaches to demographics also to be fluid, which sometimes undermines programs’ confidence that their practices are informed and adequate. Second, YES programs share a mission to diversify the cancer research workforce, though they serve particular populations of young people across the country (Appendix B). Sharing demographic approaches and challenges across YES sites through inter-grantee communication can increase awareness of approaches for measuring demographic groups more inclusively across a wider range of settings. Finally, demographic data are sensitive, and efforts to collect this information can feel invasive or value laden to trainees and programs alike. Training programs offer significant value for students; the timing and method of demographic data collection can accentuate power dynamics between the program providers and program applicants and trainees. Programs’ need for comprehensive demographic data may conflict with their obligation to preserve trainees’ trust, privacy, and well-being.

These complexities notwithstanding, a shared commitment to inclusive demographic data practices permits the identification of individuals who are being excluded, marginalized, or improperly aggregated (Fernandez et al., 2016; Morrison et al., 2021), increasing our capacity to address inequities in biomedical research training. As trainees do not enter our programs with equal access, accommodations, or preparation, inclusive demographic measures can help capture a nuanced set of program outcomes that facilitate research on intersectionality between demographic variables, including how better to retain underrepresented students in biomedical research (Duflus et al., 2014; Estrada et al., 2016; Hinton et al., 2020; Valantine et al., 2015; Valantine et al., 2016).

### Challenge 2: Operationalizing Demographics.

About half of the YES programs surveyed cited published resources that informed their demographic data practices, most commonly the Notice of NIH’s Interest in Diversity (National Institutes of Health, 2019). The notice explicitly names three demographic groups at the center of NIH diversity initiatives: (1) Racial and ethnic minorities, specifically Blacks or African Americans, Hispanics or Latinos, American Indians or Alaska Natives, Native Hawaiians and other Pacific Islanders; (2) Individuals living with disabilities, as defined by the Americans with Disabilities Act (Americans with Disabilities Act, 2009) and (3) Individuals from disadvantaged backgrounds, defined as those who meet two or more criteria including experience with homelessness or foster care, first-generation college status, eligibility for free and reduced-price lunch, Federal Pell grants, or the Special Supplemental Nutrition Program for Women, Infants and Children, and residence in a designated medically-underserved area, including rural communities. A fourth demographic, women, is mentioned in the context of intersectionality between gender and the other three categories.

The Notice of NIH’s Interest in Diversity (National Institutes of Health, 2019), along with the YES program announcement (National Institutes of Health, 2016) and NIH data reporting templates (U.S. Department of Health and Human Services, 2012) provide some guidance to YES grantees for the demographic groups to be recruited to their programs, and the demographic characteristics of trainees to be measured and reported. However, as our survey data show, programs implemented this guidance to varying degrees. Race and ethnicity were the only demographic variables collected by all responding programs (Table 1). While most programs also inquired about trainees’ disability status and socioeconomic status, less than half reported using specifically the Americans with Disability Act definitions for disability or the NIH definitions for disadvantaged backgrounds, respectively. The programs who did not list ADA or NIH left the open-field blank, so it is unclear how programs are operationalizing disability. Further, since ADA definitions describe legal protections, it is likely that middle and high school students are unfamiliar with legal considerations associated with disability. An opportunity exists for programs to collect better data while also improving how they serve students with disabilities by asking about the type of disability using the REALD instrument (McGee, 2020), which defines a series of questions that ask about functional limitations and are easily understandable to a wide range of age levels. Such questions also allow programs to align accommodations to better serve their students. However, when questions/categories are not asked, practically, this means that the operationalization of demographic categories is inconsistent across YES programs, and by extension, that data are not necessarily comparable.

Two programs cited resources offering explicit guidance on data operationalization and collection: a conference paper proposing comprehensive and inclusive approaches to demographic data, including precise wording for data collection instruments (Fernandez et al., 2016) and the Race, Ethnicity, Language, and Disability (REALD) demographic data standards (McGee, 2020). The REALD was developed for use among Oregon health care providers to identify health inequities and subpopulations requiring targeted interventions. The standards became Oregon state law for health care providers in 2020. One YES grantee and co-author of this case study adopted the REALD for use in the evaluation of students’ STEM development and biomedical research training. A public-facing website based on this work, the STEM Assessment and Reporting Tracker (Paris et al., 2021) provides definitions of demographic variables, rationale for including them in demographic data collection, and phrasing for data collection instruments. The variables include those prioritized by the NIH (i.e., race and ethnicity, disability, and disadvantaged background) as well as age, language, gender identity, and sexual orientation. For YES programs looking to refine or improve their demographic data practices, the resources cited above provide concrete, research-based models for data operationalization and best practices for data collection. Drawing from these resources and the experiences of YES programs as captured through our survey data, we offer five key recommendations (Table 4), with expanded definitions described in Appendix B. Ultimately, these considerations reflect ethical issues blending privacy and data accuracy. Programs weave these two considerations, yet there is very little guidance around best practices. For example, programs did not explicitly describe legal repercussions of demographic data collection in their responses nor did they describe required versus optional nature of students answering questions in full. With any research study, participants answering questions is voluntary, though asking questions at the time of application could increase perceived coercion. While “choose not to answer” is an answer option, it also contributes to incomplete data. One program explicity used a model that could build trust: “[s]tudents listed how they met NIH URM Criteria in free text at time of enrollment. Additionally, demographic information was collected following NIH criteria for the RPPR [NIH progress report] by email.” Thus the balance of ethics/privacy and complete/accurate data collection was a common sentiment of programs.

### Recommendation 1: Consider the Notice of NIH’s Interest in Diversity as a Starting Point for Developing Demographic Data Practices.

As described above, the notice explicitly names demographic groups prioritized by NIH diversity initiatives (including the YES program), based on research documenting the groups’ marginalization in the sciences (National Institutes of Health, 2019). At a minimum, cross-program data on trainees’ identification as racial and ethnic minorities, living with a disability, or coming from a disadvantaged background reveal in broad strokes the extent to which YES succeeds in serving these target populations. By asking specifically about trainees’ identification with NIH subcategories of disability and disadvantaged background (e.g., experiences with foster care), YES programs can increase both the degree of consistency in demographic operationalization and the alignment between trainees’ self-classifications and NIH definitions. The NIH and other funders can further support programs by operationalizing the phrasing for demographic data collection measures and disseminating data reporting tables to grantees at the onset of their award. Programs developing their own phrasing is not recommended as substantial research and sociocultural considerations are involved (McGee, 2020).

We encourage programs to expand their demographic data collection beyond the categories specified by the NIH, provided the data serve an articulated scientific or pragmatic purpose. Gender identity is one example that can lead to important insights about intersectionality with race and ethnicity, disability, and disadvantage in the context of the biomedical workforce and health inequities (Morrison et al., 2021; Paris et al., 2021; Oregon Clinical and Translational Research Institute, 2019; Suen et al., 2020). From a pragmatic perspective, data on trainees’ gender identity, including preferred pronouns, help programs address individuals appropriately and foster a safe and inclusive learning environment. Funders can facilitate advances in science and practice around health inequities and inclusive demographics by supporting collaborations between biomedical training programs and researchers (Lin, 2022).

### Recommendation 2: Increase the Granularity, or Level of Detail, of Demographic Data Collected.

Granularity can increase the validity of demographic data by creating more opportunities for respondents to select categories that accurately reflect their identities; this is especially true when respondents can select multiple options. Granularity also supports our understanding of important differences between demographic subgroups. The REALD instrument (McGee, 2020) explicitly lists 34 categories for race and ethnicity. The categories permit important research on inequities between racial and ethnic groups (McGee, 2020) yet can be easily upcoded to NIH categories for reporting (Paris et al., 2021). Further, granularity can help programs tailor materials to trainees’ needs. For example, whereas some YES programs ask trainees about disability using a single question (e.g., “Do you identify as living with a disability?”), the REALD uses as many as 10 questions, soliciting valuable information about functional limitations resulting from disability (McGee, 2020). The disaggregated data can be used to guide accommodations for individual trainees. Again, data can be up-coded for use in NIH reporting (Paris et al., 2021). Funders can encourage increased granularity by allowing the reporting of multiple identities among trainees in reporting forms and progress reports, increasing data accuracy and facilitating further research on intersectionality.

We offer two caveats. First, as granularity increases, so does the number of items and response options required for data collection, which increases the burden on respondents. There may be a limit to the number of categories a respondent is willing or able to review before becoming fatigued (Aspinall, 2009). Second, increased granularity means increased likelihood of individuals being identified through their demographic data alone, especially within smaller programs (Fernandez et al., 2016). Programs should be mindful of sample sizes when reporting to protect trainee privacy.

Intersectionality has been critically important for students in our studies (Marriott et al., 2021, 2022) and some authors found students eager to report their demographics in ways that make them feel seen. Individuals with intersectional demographics face more significant barriers and discrimination (Burns, 2013; Morton et al., 2018). Therefore, the granular data permits improved research on demographic intersectionality and which student groups are facing disparities. Without collecting such data, programs remain blind to these conditions and are at risk of perpetuating potential systems of oppression. The disaggregated ethnicity and racial characteristics explicitly address federal reporting guidelines (White House, 2022a, 2022c) which recognize that race and ethnicity are being improperly aggregated and are not taking into account the full geography and lived experiences of individuals. For example, REALD (McGee, 2020) enables populations to better describe their race and ethnicity without constraints of NIH aggregated categories that are very narrow, particularly for Asian populations. By using REALD, students have greater flexibility to accurately report their demographic backgrounds (including Tribal representation). Further, since REALD definitions can be upcoded to NIH demographic categories, with instructions explicitly defined (https://sites.google.com/view/startstem/measures/demographics), such tools can support the scientific community by offering vetted phrasing accessible to a wide age range of students and community members. Further, given that programs are trying to diversify the biomedical workforce, understanding backgrounds enables programs to improve representation of their mentors and scientists to reflect the scientific background of the students in their programs, which can support retention of historically underrepresented students (Pearson et al., 2022). Likewise, programs can use disability data to improve accommodations for students in their programs.

Many programs use online data collection platforms (i.e., Qualtrics, REDCap) for demographic data collection. These electronic survey platforms contain embedded scoring that can support easy data collection and analysis, including upcoding to NIH racial/ethnic categories for annual progress reports. It is impossible to disaggregate data that is not collected; therefore, the granular data provides substantial data for program use (e.g., representation, accommodations, aggregation for statistical analyses) that can support current and future efforts. Preliminary discussions around data granularity found that surfacing these important issues enabled programs to crowdsource gaps and solutions that improve our overall programs.

### Recommendation 3: Include Open-Response Items to Allow Trainees to Describe the Demographic Identities They Choose, in Their Own Words.

Open-response items support capture of demographic data important to trainees that may not yet be operationalized, and again provide information that allows programs to better serve individuals. Fixed-response demographic survey items can include the option, “Prefer to self-describe,” with a corresponding open field for respondents to do so (Paris et al., 2021). Surveys might also include a general open-response prompt such as, “We realize we may have not captured everything about your background or experience. If you would like to say more, please feel free to share your story” (M. Marr, personal communication, November 15, 2021). At least two YES programs have adopted this approach. As one reports, “Most trainees leave this item blank, but others take the opportunity to name their country of origin, describe the nature of a disability, add a caveat to their selections in the fixed response sections – or other meaningful details about how they identify.” Such details included a note from one trainee, who preferred non-binary gender pronouns, requesting that program personnel not “out” them to their parents. Another program defined their approach as “[s]tudents listed how they met NIH URM Criteria in free text at time of enrollment. Additionally, demographic information was collected following NIH criteria for the RPPR by email.” As NIH may be restricted in what they can ask programs to collect, open-response approaches to demographic data collection could inform new directions in inclusive demographics.

### Recommendation 4: Allow Trainees to Self-Report Their Demographic Identities.

Programs should not make assumptions about trainees’ demographic identities based on appearance, language, or other observable characteristics or affiliations. Importantly, this includes efforts to downcode, or disaggregate, high-level demographic data. Instead, self-reported data is likely to provide the most accurate demographic information (McGee, 2020). If the accuracy of self-reported data is a concern, programs may consider adopting strategies for validation or triangulation. For example, in the present survey, some YES programs noted that their trainees under-reported first-generation college status or residence within a medically-underserved community because they were unaware of the eligibility criteria (Huerta et al., (submitted); Marriott et al., (submitted)). Accuracy of data could be improved by also asking about parent or guardian educational attainment or using application addresses to verify HRSA and HPSA eligibility (Centers for Medicare and Medicaid Services, n.d.; Health Resources and Services Administration, n.d.).

### Recommendation 5: Minimize Power Dynamics.

Programs should be mindful of power dynamics created by the circumstances under which demographic data are collected. In particular, at least some demographic information is typically collected at the time of students’ application to the program, during which students may be concerned how their responses will impact their likelihood of acceptance. Programs acknowledged trade-offs with complete data collection (Table 3). Whereas basic demographic information at time of application may be required to determine program eligibility, requests for more detailed information can be delayed until greater familiarity and trust is established between programs and trainees.

### Limitations.

The matrix included in our survey was not an exhaustive list of demographic variables. Variables mentioned by some respondents through open prompt, such as age or citizenship status, are likely collected by more YES programs than represented in the data. This is especially likely with regard to citizenship status, as YES trainees must be US citizens or permanent residents to be eligible for the YES participant award (National Institutes of Health, 2016). Some demographic categories have been left out altogether (e.g., sexual and gender minorities) and highlight important reasons for training programs to communicate with scientists studying health equity and marginalized populations. While GPA was explicitly asked of programs in the context of demographic data, it was not considered a demographic variable by the study team and may have influenced programs’ responses. It does, however, provide intersectional and contextual information for how demographics and academic outcomes may be viewed.

### Future Directions.

The desire for guidance reflects how both demographics and reporting criteria have evolved (National Institutes of Health, 2016, 2019), which recognize new advancements in equitable data science and are looking for guidance in how to measure demographics to be more inclusive and accurate in their reporting. Facilitating effective comparison of data across programs is a first step toward determining the extent to which programs are collectively meeting goals for increased diversity within the cancer research workforce. While critics may argue that granular data is too much or too private, it is important to recognize that these populations are currently being marginalized in many programs with Executive Orders explicitly calling for advancing equity within data collection (White House 2021a, 2021b, 2021c; 2021d, 2022), including sexual and gender minorities and aggregated racial/ethnic groups.

The 2021-2025 NIH Strategic Plan cites “[i]mproving minority health and reducing health disparities” as one of its five cross-cutting themes (National Institutes of Health, 2021b). Diversifying the national biomedical workforce is one potentially powerful strategy to meet this goal. Our findings highlight a critical role for demographics that must be considered by cancer research training programs in an inclusive and sensitive manner. Given the interplay between advances in health inequities research, social determinants of health, and power dynamics, programs need to be mindful of populations missing from their research (Fernandez et al., 2016; Morrison et al., 2021). Our findings offer important considerations for measuring demographics needed to define and retain underrepresented populations in biomedical sciences (Boekeloo et al., 2015; Duffus et al., 2014; Valantine et al., 2015).

## Supplementary Material

Appendix A

Appendix B

## Figures and Tables

**Table 1.** Demographic-related variables collected by NCI YES programs.

Item	# Collecting Data (% respondents)	Timing of Data Collection (% collecting data)			
		At time of application	At time of enrollment	Both	Other
Race*	15/15 (100%)	11/15 (73%)	1/15 (7%)	2/15 (13%)	1/15 (7%)
Ethnicity*	15/15 (100%)	11/15 (73%)	1/15 (7%)	2/15 (13%)	1/15 (7%)
First-Generation College Status*	14/15 (93%)	10/14 (71%)	1/14 (7%)	2/14 (14%)	1/14 (7%)
Socioeconomic status (SES)*	13/15 (87%)	9/13 (69%)	0	3/13 (23%)	1/13 (8%)
Gender**	12/15 (80%)	8/12 (67%)	1/12 (8%)	1/12 (8%)	2/12 (17%)
Preferred Pronouns	10/14 (71%)	4/10 (40%)	2/10 (20%)	1/10 (10%)	3/10 (30%)
Disability status*	10/14 (71%)	8/10 (80%)	0	1/10 (10%)	1/10 (10%)
Sexual Orientation	1/14 (7%)	0	0	0	1/1 (100%)
**Other Variables**					
GPA	13/14 (93%)	13/13 (100%)	0	0	0
Language(s) spoken at home	8/15 (53%)	5/8 (63%)	0	1/8 (13%)	2/8 (25%)
**Summary Demographics**					
Explicit use of NIH (2019) criteria*	8/15 (53%)	-	-	-	-
Explicit use of race/ethnicity, disability, and SES in some form^	10/15 (67%)	-	-	-	-

Denominator reflects respondents answering that particular prompt, with 15 of 16 programs responding to the survey. Write-in responses (Appendix B) included themes of nationality and affiliations, geography, home environment, age, trainee education, and experiences.

* Demographic category specifically mentioned in the Notice of the National Institutes of Health Interest in Diversity (National Institutes of Health, 2019) or related notices that cite it, including NCI YES program announcements (National Institutes of Health, 2021a, 2022).

^ Respondents define collection of information related to racial/ethnic background, disability, and SES, though disadvantaged background’s composite variables are not explicitly described and may not match the multi-step criteria for disadvantaged background defined by National Institutes of Health (2019).

** Demographic category mentioned in the Notice of the National Institutes of Health Interest in Diversity (National Institutes of Health, 2019) in the context of intersectionality with race and ethnicity, socioeconomic status, and disability status.

**Table 2.** Evolution of YES programs’ demographic data practices over time.

Code and Subcodes	Frequency (%)	Definition	Example Quotes
**Any changes or growth in practices described**	12/15 (80%)	Programs describe any changes or rationale for changes. All programs responded to the prompt, though 3/15 (20%) explicitly described no changes to demographic items or practices	See descriptions below
**Changes to type of data collected**	8/15 (53%)	Addition or elimination of demographic items	Example quotes for sub-codes below
*Added or expanded items*	8/15 (53%)	Began collecting new categories of demographic data, or expanded response options within existing categories	“We are moving from using NIH racial/ethnic categories to using the REALD instrument (McGee, 2020) which disaggregates racial categories but can still be upcoded to NIH.”
*Eliminated items*	2/15 (13%)	Stopped collecting a category of demographic data	“Early in our recruiting process we decided to move from asking about applicants’ gender to asking their preferred pronouns instead to be more inclusive.”
**Altered data collection methods**	9/15 (60%)	Adjusted the mechanism or timing of data collection, or the wording or organization of specific data collection instruments	“We’ve moved demographic questions to the end of the application and questionnaires in an effort to limit stereotype priming, and also to communicate the relative value we place on trainees’ essays/career ambitions/etc. relative to their demographic identifiers.” “[I]mportance of collecting demographic data at the time of application to ensure complete response rate.” “We have … added descriptions of what first-generation and disability mean so students can more accurately answer those questions on the application. For example, some students may suffer from a learning disability but not realize that it would be considered as such.”
**Rationale for changes described**	9/15 (60%)	Reasons and influences for why a program evolved in their demographic changes	Example quotes for sub-codes below
*Inclusivity*	7/15 (47%)	Changes made to reflect trainees’ diverse, nuanced or intersecting identities	“We’ve added more choices in the ethnicity demographic question to better reflect the population of our region.”
*Sensitivity to social climate and inequities research*	5/15 (33%)	Changes made to reflect current perspectives on demographic categories and best practices for demographic data collection	“We now use ‘Latine’ instead of Latinx since it’s a more widely used non-binary term.” “We made the above changes to …acknowledge that terms for race/ethnicity/gender are evolving.”
*Sensitivity to trainees’ needs*	4/15 (27%)	Changes made to facilitate accommodation of trainees’ individual needs and preferences	“Languages was added… to help align students and their current skills with potential research projects that could benefit from those skills.”
*Alignment with other programs and institutions*	4/15 (27%)	Changes made to align with standards established by program funders, partners and other key stakeholders	“[W]e plan to explicitly collect demographic data required for the NIH progress report at the time of application. This was prompted by learning what data were required by NIH.”
*Accuracy*	3/15 (20%)	Changes made to improve the accuracy of data collected	“We noticed that our scholars were underreporting being first generation college students [FCGS]. Using parent’s educational attainment really bumped up students’ FCGS status.”

**Table 3.** Difficulties reported by YES programs with regard to demographic data practices.

Code	Frequency	Definition	Example Difficulties and Representative Quotes
Any difficulties in demographic practices reported by programs	11/15 (73%)	Any difficulties reported by programs. Programs who did not respond to the prompt (4 of 15, 27%) were excluded from denominator below.	See descriptions below.
Data integrity	7/11 (64%)	Concerns that collected data are incomplete or inaccurate	Knowing what to collect and how (e.g., phrasing around disability and gender identity) Incomplete data collection: “Some students do not fully fill out demographic information.” Balance between timing of data collection for optimal response rate and comfort level of trainees (e.g., sensitive demographic data often asked in the context of trainee application). Accuracy of trainee self-report data: “We take trainees at their word regarding their identity with the demographic categories included in our survey. I worry this is insufficient - not so much that trainees are being dishonest, but rather that they have different interpretations than our team, and especially that they would be considered first-gen/economically disadvantaged by NIH and not realize it.” Alignment across data sources (e.g., international 4-year degree equivalents; (Tse, 2012; UNESCO, 2004): “How to define first generation college status for immigrant families who received degrees in other countries.”
Protection of trainee privacy and dignity	5/11 (45%)	Concerns that the practice of data collection may negatively impact trainees, specifically with regard to their sense of privacy or self-worth	Tension between inclusivity, comprehensiveness of data, and student privacy: “Our main problem is …trying to collect as much data while also respecting privacy. We do allow applicants/participants to not answer any question that they do not want to answer. However, they have to answer at least one of the questions above to qualify for the program. So there is a chance of lost data.” Concerns for trainee emotional wellbeing: “Data is collected during outreach efforts that can identify students living in troubled neighborhoods (i.e., higher crime rates, food deserts, poverty stricken areas, etc) through conversations with teachers, program directors, and principals. Students have no desire to be known as a have not…so we don’t embarrass them by making them answer these types of questions.” Concerns over insufficient bandwidth to analyze collected data: “I feel like we shouldn’t ask for personal information we don’t use. However, our team is guilty of that. Parent/guardian employment info is one example. … We haven’t had time to analyze it.”
Data analysis and reporting	4/11 (36%)	Concerns regarding practical or sociopolitical implications of practices in data analysis and reporting; responsiveness of programs to data	Consistency/comparability of data from year to year: “The discussions [regarding demographic categories, such as gender identity] are ever evolving, and continually modifying survey questions can lead to difficulty reconciling data from year to year.” Reconciliation of data needs across stakeholder groups:“It would be helpful at the time of funding what data would be needed for reporting.” “The NIH reporting that we’re required to do is problematic - it asks programs to group students identifying as more than one race into that bucket (labeled ‘mixed race’ in most recent document requests). This underreports racial/ethnic groups in our dataset.”
Data utility	3/11 (27%)	Concerns regarding pragmatic value of data (i.e. for program logistics, trainee experience, or outcomes)	How to be responsive to data to best serve trainees: “We currently don’t collect [data on disability status] and it’s not an eligibility criteria for our program but we also want to be able to best support any students who we serve. Educating trainees about demographic categories and aligning understandings: “Students are not aware that they are first generation college or come from a disadvantaged background. Many are unaware that they are considered historically underrepresented.” Use of data by organizational systems: “Asking for pronouns rather than specific gender has sometimes made it difficult to gauge whether or not we might have even numbers of male and female students. This is only an issue when it comes to our high school summer residential program when we have to follow housing guidelines on campus.”

**Table 4.** Practice recommendations for collecting demographic data (expanded definitions in Appendix B).

Target Audience	Key Recommendations for Inclusive Demographics
**Training programs**	1. Consider the Notice of NIH’s Interest in Diversity as a starting point for developing demographic data practices. Expand data collection as scientific and pragmatic needs dictate.
	2. Increase granularity of demographic data to improve data accuracy and understanding of important differences between demographic subgroups.
	3. Include open-response items for trainees to describe the demographic identities they choose, in their own words, to increase data accuracy and support emerging research. Include prompts for pronouns and sexual and gender minorities (Morrison et al., 2021).
	4. Allow trainees to self-report demographic identities to increase data accuracy; Adopt strategies for data validation as needed to limit underreporting.
	5. Minimize power dynamics through sensitivity to the circumstances under which demographic data are collected.
**Funders**	1. Operationalize phrasing for demographic data collection measures, and disseminate data reporting tables at onset of award, to increase consistency in data collection across programs.
	2. Support collaborations between biomedical training programs and researchers to facilitate advances in science and practice around health inequities and inclusive demographics.
	3. Permit reporting of multiple identities among trainees to increase data accuracy and permit further research on intersectionality.
	4. Expand considerations for sexual and gender minorities (White House, 2021a, 2022)

## ABBREVIATIONS

ADA: Americans with Disability Act
GPA: Grade Point Average
IRB: Institutional Review Board
NCI: National Cancer Institute
NIH: National Institutes of Health
REALD: Race, Ethnicity, Language, and Disability
SES: Socioeconomic Status
STEM: Science, Technology, Engineering and Mathematics
YES: Youth Enjoy Science
