# Supplementary material for: Approaches for Measuring Inclusive Demographics Across Youth Enjoy Science Cancer Research Training Programs
Source: J STEM Outreach. Author manuscript; Available in PMC 2023 Oct 13. (PMC10575593; doi:10.15695/jstem/v5i2.12)
Supplement: Appendix A [file NIHMS1893168-supplement-Appendix_A.pdf]

Approaches to Inclusive Trainee Demographics – Mekinda et al.
Appendix A. Survey

Survey sent to YES grantees

Intro text: The Harmonizing Trainee Surveys group is drafting a paper on the collection, interpretation and use of trainee demographic data across NCI R25 YES programs. We need your input! Please provide the information requested below to strengthen our review and recommendations.

- Name
- Institution
- What is your role with regard to your NCI R25 YES program? [Fixed response: Principal Investigator, Administrator, Other (please specify)]
- In what year was your NCI R25 YES grant first awarded? [Open response]
- Please indicate if and when you collect the following demographic information for your NCI R25 YES trainees: Race, Ethnicity, Gender, Preferred pronouns, Sexual orientation, First-generation college status, Socioeconomic status, Disability status, Language(s) spoken at home, Grade point average [Fixed response: At time of application; At time of enrollment; Not collected; Other (please specify)]

<u>If gender data collected</u>
In collecting information about trainees' GENDER, do you include non-binary options (i.e., gender identities other than "male" or "female")? [Fixed response: Yes, No]

<u>If first-generation college status data collected</u>
In collecting information about trainees' FIRST-GENERATION COLLEGE STATUS, do you ask about parents' educational attainment? [Fixed response: Yes, No]

<u>If socioeconomic status data collected</u>
In collecting information about trainees' SOCIOECONOMIC STATUS, do you ask about criteria outlined in the Notice of NIH's Interest in Diversity (i.e., homelessness, foster care, free/reduced-price lunch, parents' educational attainment, Pell grant eligibility, Special Supplemental Nutrition Program eligibility, or residence in a rural or medically-underserved area)? [Fixed response: Yes, No]

<u>If disability status data collected</u>
In collecting information about trainees' DISABILITY STATUS, do you ask about criteria referenced in the Notice of NIH's Interest in Diversity (i.e., the nature of the disability or the extent to which it impacts major life activities)? [Fixed response: Yes, No]

- Please list any demographic data collected by your team not included in the list above. [Open response]
- Please describe any documentation you collect to crosscheck trainee demographic information (e.g., academic transcripts to verify trainees' GPA or attestation from a financial aid office to verify trainees' socioeconomic status). [Open response]
- Of the demographic data collected at time of application, which categories factor into your team's admissions decisions? [Open response]
- How do you collect demographic information for MINORS enrolled in your program? [Fixed response: Participant self-report, Parent/guardian report, Combination of self and parent/guardian report, Not applicable]

- Please describe any ways your team's approach to collecting, interpreting or responding to trainees' demographic information has evolved over the course of your YES program. For example, have you added or deleted items from your demographics questionnaire? Have you made changes to specific language or word choice? What prompted these decisions? [Open response]
- Please describe any questions or difficulties your team currently has regarding the collection, interpretation or application of trainees' demographic information. [Open response]
- If you are willing, please upload a copy of your trainee demographics questionnaire to best inform our review and recommendations. [Document upload]
- Please share citations or links for resources that have informed your demographics questionnaire (e.g., the Notice of NIH's Interest in Diversity). [Open response]
- Please use this space to share any additional thoughts on demographic data collection, interpretation and use that the Harmonizing Trainee Surveys team should consider in preparing our review and recommendations. [Open response]
- For approximately how many years have you been involved in training programs to serve historically underrepresented students? [Open response]
