# Supplementary material for: Approaches for Measuring Inclusive Demographics Across Youth Enjoy Science Cancer Research Training Programs
Source: J STEM Outreach. Author manuscript; Available in PMC 2023 Oct 13. (PMC10575593; doi:10.15695/jstem/v5i2.12)
Supplement: Appendix B [file NIHMS1893168-supplement-Appendix_B.pdf]

Approaches to Inclusive Trainee Demographics – Mekinda et al.
Appendix B. Qualitative Results.

Coding Dictionary

Demographic data collected by programs

Table B1. Demographic-related variables in open-ended prompts of YES programs.

Code	Definition	Examples Provided by Programs (frequency reported)
Nationality and Affiliations	Programs describe affiliation with specific groups or countries, including tribal affiliations, citizenship, permanent resident status, and refugee/immigrant status	Citizenship status (n=2), permanent resident status; Refugee/immigrant status; Tribal affiliation (n=2); Veteran's status (asked of undergraduates)
Geography	Geographical location including zip code, mailing address, or characteristics describing area, such as urban-rural-frontier designation (Health Resources and Services Administration, 2012; Oregon Office of Rural Health, 2019)	Zip code (n=2), mailing address, urban-rural-frontier area or community
Home environment	Environment in which the trainee is growing up, including single parent/guardian home, parent/guardian employment, and medical-related employment status	Single parent/guardian home; medical-related employment status, parent/guardian employment
Age	Includes age or date of birth	Date of birth/age (n=2),
Education Status	Includes a range of items that describe educational status of trainee, including summaries of grade point average (GPA), grades, courses taken, school enrollments, and classifications. Transcripts were also included within education status.	Updated transcripts/grades to verify GPA; Education status of college student (classification/classes taken);<br>high school attended; college enrollment
Experiences	Describes extra-curricular or personal experiences that may influence a trainees understanding of cancer including its impact	Family history of cancer (via essay), outreach activities (via essay)

** In post-hoc analyses, demographic variables were discussed in context of geographical focus of programs. For example, one program cited that the REALD instrument (McGee, 2020) didn't include "Cuban" as one of the racial/ethnic categories answer options, though it could be described in open-ended prompts. The program cited that additional granularity and flexibility may be important for a program based in Miami. Likewise, Tribal affiliation may be essential for a program in Nebraska, but less so for Chicago's south side. There is a balance between mandating specific demographic categories and permitting flexibility for programs to accommodate their particular trainee populations.*

Data verification reported by programs

Table B2. Verification of trainee information by YES programs responding to prompt "Please describe any documentation you collect to crosscheck trainee demographic information (e.g., academic transcripts to verify trainee's GPA or attestation from financial aid office to verify trainees' socioeconomic status)"

Code	Frequency (% of programs)	Definition	Example Quote
Any verification	14/15 (93%)	Any sort of verification is reported by programs, regardless of type of information (e.g., non-demographic).	"Transcripts for GPA"; "Students must be in "good standing." We verify academic status with transcript - but do not focus on exact GPA - everything else is self-reported"
No verification	1/15 (7%)	No verification processes are used.	"None."
Demographic verification	3/15 (20%)	Programs describe approaches for verifying demographics defined in NIH Interest in Diversity (National Institutes of Health, 2019): race/ ethnicity, disability, and disadvantaged backgrounds.	Note: each program reporting verification of demographic information used a different approach.
Parental attestation	1/15 (7%)	Programs used parent confirmation of trainee's application for accuracy.	"For demographic information, we make parents sign a form attesting to the truth of what the student wrote. We do have language stating that we can ask for more. This is largely to protect against those gaming the system."
Affiliation	1/15 (7%)	Programs verified enrollment or eligibility with specific groups, such as eligibility in statewide programs that serve racial/ethnic groups	"Enrollment based on eligibility for [State's] Public Schools' Title VI Indian Education-funded Native Indigenous Centered Education program"
Financial	1/15 (7%)	Approach to verify disadvantaged background of students based on socioeconomics (SES).	"Optional submission of redacted tax forms, FASFA forms, or other documentation that demonstrate low SES status"
School transcripts	12/14 (86%)	Programs verified grade point average (GPA), course grades, and school enrollment using official or unofficial transcripts. Changes in approach describing transcripts included.	"We collect updated transcripts and/or grade reports for applicants as part of their application to verify GPA which is self-reported on the application."
Self-report information	3/15 (20%)	Programs describe that they use self-report information from their trainees, either at the time of application or enrollment.	"We used to collect transcripts, but that was a large burden for trainees and moved to asking GPA instead"
Triangulation of sources	2/15 (13%)	Programs describe getting a better understanding of applicants through interviews or letters of recommendation	"We collect baseline data as part of the application. We interview all applicants and ask the questions during the interview and explore them further as needed to assure accuracy of our data."<br>"Academic transcripts and two letters of recommendation. (one from HS guidance counselor and one from science teacher)."

Admissions data collected by YES programs

Table B3. Demographic-related variables used for admissions decisions by YES programs based on the prompt "Of the demographic data collected at time of application, which categories factor into your team's admissions decisions?"

Code	Frequency	Definition	Example Quotes
Diversity	11/15 (73%)	Describes underrepresented factors defined in NIH criteria (2019), including race/ethnicity, disability, or disadvantaged background (e.g., first-generation college status, socio-economics). Includes program reference to using all [categories].	• "Race/ethnicity, first-generation college status, and socioeconomic status"<br>• "Meeting the NIH URM Criteria"<br>• "All that are collected."<br>• "We seek to achieve a diverse cohort of learners based on race, ethnicity, first generation, low income, gender, and other non-socioeconomic factors that will assure a diverse group of undergraduate participants, e.g., matriculating freshmen vs. community college transfer students"
Target populations or service area	4/15 (27%)	Key populations or target areas described in the context of diversity and demographic factors used for program reach that are defined outside the context of admissions decisions.	• "We only consider these categories to see if students qualify (if we can serve them), but they play no other factor in our admission decision."<br>• "As long as the students are eligible, we do not factor the demographic data into admissions decisions. However, we do have automatic admission for a few close community partners and their students. For example, we will accept: 1) Deaf and Hard of Hearing Students each year, 2) students from Funding for the Advancement of Minorities through Education (predominately Black students), 3) Students from the Homeless Children Education Fund (all experience homelessness), etc. We do monitor the gender, racial, and ethnic breakdown of students before sending acceptance letters to make sure there is a balance, however, we rarely make changes because of this."<br>• "Data is collected during outreach efforts that can identify students living in troubled neighborhoods (i.e., higher crime rates, food deserts, poverty-stricken areas, etc.) through conversations with teachers, program directors, and principals. Students have no desire to be known as a have not... so we don't embarrass them by making them answer these types of questions."
Non-demographic items	5/15 (33%)	Grades, student essays, interviews, and letters of recommendation are described as being factored into decisions in the presence or absence of demographic data. Also includes change in collection procedures.	• "The primary factors are the interview, essay submitted, followed by GPA, and then by diversity."<br>• "GPA was removed from admissions decisions based on the pleading of our teacher advisory team. They felt it was inequitable due the unconscious bias that can go into student grading in the first place. Plus, some students have competing demands (family/work obligations) that can interfere with schoolwork due to SES issues more than ability to do the work."

Resources for demographic data collection provided by YES programs

Table B4. Resources described by YES programs for informing demographic data collection.

Code	Frequency	Definition	Cited Examples
Any resources	8/15 (53%)	Program provided examples of resources used by their programs	Described below
NIH Notice of Diversity	7/15 (47%)	NIH Notice of Diversity or NIH webpage on diversity were specifically cited.	• NIH Diversity https://diversity.nih.gov/about-us/population-underrepresented<br>• https://grants.nih.gov/grants/guide/notice-files/NOT-OD-20-031.html
Specific resources	4/15 (27%)	Other examples provided outside of NIH Notice of Diversity	One program described the YES program announcement, with other resource categories described below.
Race/ethnicity	1/15 (7%)	Race/ethnicity resources described outside the context of NIH Notice of Diversity (National Institutes of Health, 2019)	REALD - Race/Ethnicity, Language, Disability Instrument from Oregon Health Authority (McGee, 2020) https://www.oregon.gov/oha/OEI/Pages/REALD.aspx
Disability resources	3/15 (33%)	Resources for collecting information about disability	• Disability described by ADA https://www.ada.gov/pubs/adastatute08.htm<br>• REALD describing functional limitations (McGee, 2020)
Socioeconomics	2/15 (13%)	Resources for collecting information about trainee socioeconomics	Income Guidelines: https://www2.ed.gov/about/offices/list/ope/trio/incomelevels.html
Literature and tools provided	2/15 (13%)	Other citations and tools used to inform practices and educate others	• Inclusive demographics (Fernandez et al., 2016) https://docs.lib.purdue.edu/cgi/viewcontent.cgi?article=1059&context=enegs<br>• Demographics in the context of career expectancy (Metz, Fouad, and Ihle-Helledy, 2008) https://journals.sagepub.com/doi/pdf/10.1177/1069072708328862<br>• STEM Assessment and Reporting Tracker - NIH-funded website offering approaches for inclusive measurement of demographics for STEM and biomedical training programs (https://sites.google.com/view/startstem/measures/demographics)
Nothing described	7/15 (47%)	Program left item blank or described N/A	"N/A"

Secondary analysis of themes provided by YES programs

Table B5. Practice recommendations for collecting demographic data.

Consideration	Rationale for Practice Decisions
**Self-report data is inclusive**	One program recommended REALD, which provided compelling rationale for using self-report (McGee, 2020) because a key principle underlying REALD is that of self-reporting. State administrative rules specify that the "Authority, Department, or Contractor shall not assume or judge ethnic and racial identity, preferred signed, written and spoken language, or disability without asking the individual" (OAR 943-070-0200(3)(a)). Since REALD data standards reflect identities, language preferences and functional limitations, self-reporting will typically provide the most accurate information (Bilheimer & Sisk, 2008; Hasnain-Wynia & Baker, 2006; Kressin, Bei-Hung, Hendricks, & Kazis, 2003; Schmidt et al., 2015; Ulmer et al., 2009). REALD emphasized that it is also important to avoid making assumptions about the person based upon shared membership in a certain community (McGee, 2020).
**Open response options can advance inequities research**	• New research has highlighted approaches that support accurate identification and inclusive data collection for sexual and gender minorities (Morrison, Dinno, and Salmon, 2021; OHSU Evaluation Core, 2019; STEM Assessment and Reporting Tracker, 2021; Suen et al., 2019), such as using open prompts when asking about preferred pronouns or sexual and gender identity. If fixed responses are presented, programs should include answer options beyond the gender binary without use of "other" in answer options (STEM Assessment and Reporting Tracker, 2021).<br>• To support capture of demographics important to trainees that may not yet be operationalized, one approach could be including an open-ended prompt that allows trainees to self-report additional demographics and identities that may be missing (e.g., "We realize we may have not captured everything about your background or experience. If you would like to say more, please feel free to share your story"; Marr, 2021). As NIH may be more restricted in what they are able to ask programs to collect, such approaches could inform new directions in inclusive demographics.
**Granularity encourages better understanding of populations**	• For fixed-response items, programs should consider increasing the granularity, or level of detail, of data collected. The REALD (McGee, 2020), for example, calls for a degree of granularity that far exceeds the Notice of NIH's Interest in Diversity (National Institutes of Health, 2019). The REALD instrument explicitly lists 34 categories for race and ethnicity alone, with a caveat that more categories may be required as additional populations emerge. These categories permit important research on inequities yet still be easily upcoded to NIH categories for reporting (START, 2021). Collecting high-level demographics does not support downcoding (i.e., data disaggregation).<br>• Granularity supports accuracy around multiple identities. Participants should be allowed to select multiple identities as needed (i.e., racial/ethnic). Granularity provides opportunities for programs to use disaggregated data to tailor materials and interpret findings. Programs described that demographic data informed how programs were structured to best serve trainees.<br>• As granularity increases, the number of questions may increase, which increases time needed to complete surveys. For example, some YES programs ask trainees about disability using a single question whereas the REALD uses 2-10 questions to identify functional limitations that can be upcoded to NIH disability (START, 2021). Functional limitation questions permit programs to understand accommodations needed.<br>• Smaller programs reported concerns about identification of students with granular data. Programs should be mindful of sample sizes in reporting, as privacy concerns emerge as granularity increases. The ethical balance between accuracy and privacy needs to be considered in decisions.
**Validation can support accuracy and trainee education**	• Programs can validate trainees' eligibility for a given demographic category in the manner they choose, or not to validate at all. Verification of some measures can be helpful, not only for accuracy of data collection, but also for trainees. Programs described first-generation college status and rural eligibility were underreported in their programs, which increased when trainees were asked for their parents/guardians' educational attainment or rural data was verified using address/zip code (Huerta et al., 2022; Marriott et al., 2022), highlighting that many trainees may be unaware that they qualify for important scholarship eligibility metrics.<br>• Non-demographic items were also requested of participants for contextual information. As cited by one program, "[m]any of our students are from low-income families whose financial stability is precarious from day to day. We have been trying to figure out a way to capture how this impacts the students' chances of persisting in college through to graduation and matriculation into graduate school." Such considerations may inform why non-demographic items like GPA are often included, as it permits research on intersectionality between demographics and academic outcomes important for career expectancy (Metz, Fouad, and Ihle-Helledy, 2008). Some programs validated GPA against transcripts.
**Reduce power dynamics when possible**	Programs should be mindful of power dynamics, student privacy, and contexts for when demographic questions are asked (e.g., in context of admissions). Students may be more hesitant to disclose certain demographics at the time of application (e.g., learning differences). Programs must balance when they ask demographics questions with program eligibility criteria, since they often overlapped.

Table B6. Recommendations for funders to improve consistency of demographic data collection.

Guidance Needed	Rationale
**Operationalizing demographic phrasing**	• Programs cited uncertainty around operationalizing demographics, particularly across gender identity, sexual orientation (i.e., LGBTQ+ referring to lesbian, gay, bisexual, transgender, and queer trainees), disability, and disadvantaged backgrounds.<br>• Operationalizing wording for disability and disadvantaged background questions is particularly needed since both are used to define underrepresented populations in biomedical science (National Institutes of Health, 2019). Programs developing their own phrasing is not recommended as substantial research and sociocultural considerations are involved (McGee, 2020).<br>• Programs want to support NIH and were interested in desired fields for reporting tables. Some suggested that these tables or recommended phrasing should be shared with grantees and potential grantees to support inform practice around consistent data collection across categories.
**Reporting Disaggregation**	Reporting documents to NIH could be improved by permitting selection of multiple racial/ethnic identities among students (instead of grouping to more than one race), which accurately reflects identities, minimizes underreporting of racial/ethnic categories, and permits further research on intersectionality. Programs described upcoding demographics to fit NIH reports, which felt incongruent with demographics collected.
**Encourage links with inequities researchers**	• Programs appreciated that science around health inequities and inclusive demographics was advancing. Supporting links between biomedical training programs and health inequities researchers who can inform practice could advance research in both areas.<br>• Guidance around how to align data collection approaches over time is needed.
